# Availability, diversification and versatility explain human selection of introduced plants in Ecuadorian traditional medicine

**DOI:** 10.1371/journal.pone.0184369

**Published:** 2017-09-08

**Authors:** G. Hart, Orou G. Gaoue, Lucía de la Torre, Hugo Navarrete, Priscilla Muriel, Manuel J. Macía, Henrik Balslev, Susana León-Yánez, Peter Jørgensen, David Cameron Duffy

**Affiliations:** 1 Department of Botany, University of Hawai‘i at Mānoa, Honolulu, Hawai‘i, United States of America; 2 Department of Geography, Environmental Management and Energy Studies, University of Johannesburg, APK Campus, Johannesburg, South Africa; 3 Herbario QCA, Pontificia Universidad Católica del Ecuador, Quito, Ecuador; 4 Escuela de Biología, Pontificia Universidad Católica del Ecuador, Quito, Ecuador; 5 Departamento de Biología, Área de Bótanica, Universidad Autónoma de Madrid, Madrid, Spain; 6 Institut for Bioscience, Aarhus Universitet, Aarhus, Denmark; 7 Missouri Botanical Garden, St. Louis, Missouri, United States of America; 8 Pacific Cooperative Studies Unit, University of Hawai‘i at Mānoa, Honolulu, Hawai‘i, United States of America; Oklahoma State University, UNITED STATES

## Abstract

Globally, a majority of people use plants as a primary source of healthcare and introduced plants are increasingly discussed as medicine. Protecting this resource for human health depends upon understanding which plants are used and how use patterns will change over time. The increasing use of introduced plants in local pharmacopoeia has been explained by their greater abundance or accessibility (*availability hypothesis*), their ability to cure medical conditions that are not treated by native plants (*diversification hypothesis*), or as a result of the introduced plants’ having many different simultaneous roles (*versatility hypothesis*). In order to describe the role of introduced plants in Ecuador, and to test these three hypotheses, we asked if introduced plants are over-represented in the Ecuadorian pharmacopoeia, and if their use as medicine is best explained by the introduced plants’ greater availability, different therapeutic applications, or greater number of use categories. Drawing on 44,585 plant-use entries, and the checklist of >17,000 species found in Ecuador, we used multi-model inference to test if more introduced plants are used as medicines in Ecuador than expected by chance, and examine the support for each of the three hypotheses above. We find nuanced support for all hypotheses. More introduced plants are utilized than would be expected by chance, which can be explained by geographic distribution, their strong association with cultivation, diversification (except with regard to introduced diseases), and therapeutic versatility, but not versatility of use categories. Introduced plants make a disproportionately high contribution to plant medicine in Ecuador. The strong association of cultivation with introduced medicinal plant use highlights the importance of the maintenance of human-mediated environments such as homegardens and agroforests for the provisioning of healthcare services.

## Introduction

Understanding how people select plants for medicinal use has been a central question in ethnobotany [1]. Early ethnobotanical studies showed that people use a large proportion of available plant species [2]. The number of medicinal plants used by a community or region generally increases with the species richness of the local flora [3], and is neither random nor temporally static. Particular plant families may be more or less well represented out of proportion to their abundance [4–6], and species use will change over time because of cultural, economic, political and ecological influences [7]. To better understand the process of plant selection, it is important to explore how and why plant species are accepted and brought into use by a community.

In the majority of countries in the developing world, 70–95% of people rely on plant medicine as a primary source of healthcare [8]. Introduced plant species have a long history in these medical systems, including cosmopolitan cultivars (e.g. ginger, *Zingiber officinale* Roscoe), naturalized species (e.g. plantain, *Plantago major* L.) and commercially traded species (e.g. wormwood, *Artemisia annua* L.). In some regions, the use of introduced species in traditional medicine is reported to be increasing (e.g. [9]). Introduced plants are defined here as plants that arrived to a specified region through the direct or indirect aid of humans. Introduced species, particularly those that are weedy or invasive, are typically seen as major ecosystem change agents and threats to biodiversity in the broader conservation biology literature [10,11]; while for traditional healers and other cultural practitioners, introduced plant species may have a strong cultural importance [12–15]. Several studies report the importance or widespread medicinal use of introduced, exotic or alien plant species [7,12,16–22], invasive species [13,14,23], and weedy species and their associated habitats [24–28]. The use of introduced plants as medicine should not be surprising, as pharmacopoeia are dynamic and healers can be expected to experiment with plants in the environment regardless of their origin [7]. However, it has been debated as to whether an increase in the use of introduced plants as medicine presents a concern for the protection of traditional knowledge and plant-based healthcare (i.e., when non-native plants replace the use of natives) [29]. Assessing these potential impacts depends upon understanding the patterns of change over time and the relative importance of drivers behind this change [29].

Three main hypotheses have been suggested to explain the incorporation of introduced species into a given pharmacopeia. The *availability hypothesis* states that the greater accessibility or abundance of introduced plants compared to native plants explains their incorporation or value as medicine [26,29]. The greater availability of introduced species to people is among the most commonly cited reasons for their use or importance as medicines [19]. Availability is often conceptualized as a physical distance from a home or community to the location where a plant grows in the wild, but could also be considered in terms of price, as well as access to markets or gardens [30]. Though not typically discussed in papers that test the availability hypothesis, plant cultivation in homegardens or areas accessible to traditional healers and others who use medicinal plants would also increase availability.

Second, the *diversification hypothesis* suggests that introduced species fill therapeutic vacancies, perhaps due to novel bioactivity, thereby diversifying the set of treatment options [20,29,31]. The diversification hypothesis has been explored by examining the novelty of introduced plant species both through interviews with healers and through chemical assay [20,31], but could also be considered in terms of plants’ providing treatment options for newly introduced diseases [32]. Diversification has been presented as an alternative to the idea that traditional knowledge is lost with the increased use of introduced plants for medicine [29].

Third, the *versatility hypothesis* suggests introduced plants are more likely to be incorporated as medicines because they have a wider range of uses and/or can treat a wider range of diseases and symptoms than native plants [16]. Versatility typically refers to the number of body systems or the number of diseases or symptoms a plant treats. In some cases, introduced species have been found to be the more versatile than native species [16]. Versatility may also refer to the number of use categories for a plant (food, medicine, technology, ceremony, etc.). Palms (Arecaceae), for example, are the most useful tree family in some regions according to this criterion [2].

While these hypotheses are not mutually exclusive, they are often tested independently. In this study, we attempt a robust test of these three hypotheses as alternative or complementary mechanistic explanations for the inclusion of introduced plant species in the Ecuadorian pharmacopoeia. We approach the issue by evaluating the support for multiple, non-independent hypotheses simultaneously, similar to a model selection process. We utilize the extensive ethnobotanical information contained in *the Encyclopedia of Useful Plants of Ecuador* [33] and floristic data from *The Catalogue of Vascular Plants of Ecuador* [34] to explore the representation of introduced medicinal plants in Ecuador, and to quantitatively examine the three hypotheses mentioned above. We aim to answer the following questions: 1) Are introduced species over-represented in the Ecuadorian pharmacopoeia?, and if so, 2) Is this over-representation best explained by their: a) greater availability, b) different therapeutic applications?, or c) greater number of categories of use? This paper contributes to our understanding of how people select plants for medicine and provides information concerning the use of introduced species that is expected to be useful in medicinal plant and traditional knowledge conservation planning.

## Methods

### The databases

We used a compilation of ethnobotanical data (44,585 entries) contained in *the Encyclopedia of Useful Plants of Ecuador* [33], hereafter referred to as *the Encyclopedia*, to investigate the representation of introduced plant species in the Ecuadorian pharmacopoeia. This encyclopedia draws from a collation of ethnobotanical research that has been carried out in Ecuador since the 18^th^ century in at least 100 communities and 14 ethnic groups. The data were compiled from previously published work (154 bibliographic references) and from over 19,000 herbarium specimens [33]. In addition, when referring to the overall Ecuadorian flora, we used floristic data from *The Catalogue of Vascular Plants of Ecuador* (Jørgensen and León-Yánez 1999), hereafter referred to as *The Catalogue*. Of the more than 17,000 recorded vascular plant species in Ecuador [34,35], 5,172, or approximately 3 out of 10, plants have recorded human uses, with 60% of those being medicinal [33].

In this paper, in order to provide the most comparable units for analysis, we included entries from our two sources at the species level, excluding subspecies and varieties. We also excluded species from analysis whose native or introduced status could not be determined. For analyses that included geographic distribution, we also excluded species in *the Encyclopedia* that could not be matched with a species name in *The Catalogue*. A complete list of introduced medicinal plants analyzed in this study with cultivation status can be found in the supporting information (S1 Table). Cultivation status followed designations in *The Catalogue*. All introduced medicinal species names (S1 Table) were updated for the supporting information according to www.theplantlist.org. We also assessed the likelihood that taxonomic revisions since the publication of *The Catalogue* would influence our findings. Out of 312 introduced plants utilized in medicine, only ten (3.2%) had been reassessed as synonymous with another species in our study, or had been reduced to a subspecies and, therefore, would no longer be included in our study. Out of 300 randomly selected native medicinal plants, none had been reassessed as synonymous with another species in our study, or had been reduced to a subspecies.

### Representation of introduced plants as traditional medicine

To determine whether plant origin (introduced or native) helps to explain the selection of a plant for medicinal use in Ecuador, we created a 2×2 contingency table representing plant origin (introduced or native) and plant status as a medicinal or not. A Pearson’s Chi-squared test with Yate’s continuity correction was used to test the hypothesis of non-independence between plant origin and use as medicine.

To understand the magnitude of potential differences in representation of introduced and native plants in the pharmacopoeia, we compared the proportion of introduced species used as medicine [33] to the total proportion of introduced species in the overall flora of Ecuador [34,35]. We did not include the 186 species listed by [34] as expected to be documented in Ecuador in the future, as we do not have information on their current distribution.

### Availability hypothesis: Accessibility of plants for use

To test how well availability factors predict the use of introduced species as medicine, we estimated plant availability as the number of Ecuadorian governmental provinces in which the plant occurs, and by the plant’s cultivation status. The number of provinces of occurrence was used as a proxy for availability because it provides a measure of how widespread each plant is within the country. It was calculated from data in *The Catalogue* [34] (S2 Table). Species with a wider distribution can be expected to be available to a larger number of traditional healers, leading to an increased likelihood of medicinal use. While we would prefer information on plant abundance or local commonness, data is available only for distribution by province, which we therefore include as a coarse estimate of availability. We also considered cultivation status as a measure of availability because cultivation can be expected to increase plant accessibility through direct human manipulation. We evaluated our model with and without cultivated species to better understand the role of cultivation in shaping our findings.

We used a generalized linear model with binomial error structure to test if the status of a given plant as medicinal (response variable) was dependent on its availability (number of provinces of occurrence and cultivation status) and its origin (introduced or not). This error structure was selected given the binary data for the response variable. Plant origin was included into the model to explore its interaction with the number of provinces of occurrence. A significant interaction between plant origin and number of provinces of occurrence would suggest that the availability of introduced plants has particular significance for their use as medicinals. The continuous predictor (number of provinces of occurrence) was standardized to facilitate comparison with other predictors. Direct comparison of the number of plants in cultivation and number of provinces of occurrence were made between introduced and native plants for the flora overall, and for medicinal plants in particular, with Pearson’s Chi-squared test with Yate’s continuity correction and with a Kruskal-Wallis rank sum test.

### Diversification hypothesis: Therapeutic applications

To test the hypothesis that introduced species provide different therapeutic applications than native medicinal plants, we compared the therapeutic application of introduced and native medicinal species in three ways. First, we calculated the number of native and introduced species that fall into each medicinal treatment category, based on the body system and the disease categories in *the Encyclopedia* (modified from [36]). We then used log-linear analysis and multi-model inference [37] to see if the species’ origin (introduced or native) made a significant contribution to explaining the number of species in each treatment category. We did this by comparing a generalized linear model with and without the interaction between origin and treatment category (or body system).

Second, we compared the number of species of native and introduced plants used to treat diseases introduced to the New World (i.e., post-Contact Old World diseases such as smallpox, measles, malaria, influenza and chickenpox) and those already present in South America prior to Contact, using a 2×2 contingency table and a Chi-square test. This comparison indirectly tested if species may have been introduced to the pharmacopoeia to diversify options and fill a therapeutic vacancy. In order to designate plant species as treating Old or New World diseases, we categorized plant use records through newly created search criteria to provide information about the particular disease a plant treats. We included all diseases and syndromes (not symptoms) that could be identified as either New or Old World in origin (S3 Table). Disease origin for smallpox, measles and cholera was determined based on [38], p18, for leishmaniasis based on [39], and for mumps based on [40]. Tuberculosis and syphilis were not included because their origin is considered unresolved [40].

Third, we calculated a medicinal redundancy score (the inverse of uniqueness) for each plant, which was the average number of additional plant species recorded as treating the same conditions that the plant treats. To calculate this, we used the same search criteria as mentioned above (S3 Table). A Wilcoxon Rank Sum Test was used to compare the redundancy of introduced and native medicinal plant species. Finding that introduced species have a lower redundancy (higher uniqueness) would support the diversification hypothesis by suggesting the introduced plants fill vacancies or conditions with fewer treatment options.

### Versatility hypothesis: Number of plant uses

To determine if introduced useful plants had a larger number of uses than native useful plants, we calculated the number of use categories for each plant (e.g., medicine, food, fuel), and the number of medicinal treatment categories (e.g., antidote, circulatory system disorders) according to categories in *the Encyclopedia*. Differences between introduced and native plants were evaluated using a Kruskal-Wallis test for both comparisons. These, and all statistical analyses, were performed in R [41].

## Results

### Representation of introduced plants in the pharmacopoeia

We reject the null hypothesis that plant origin (introduced or native) is independent of its use for medicine (χ^2^ = 203.48 df = 1, p < 0.0001; Table 1). The proportion of introduced species in the Ecuadorian pharmacopoeia is approximately four-times their proportion in the flora overall (Table 1). Introduced species are over-represented in the pharmacopoeia. They make up 12.3% (312 of 2541) of the total species in the Ecuadorian pharmacopoeia as documented in *the Encyclopedia*, while accounting for only 3.7% (595 out of 15,901) of the documented plants in Ecuador [34,35]. Out of the 20 most-mentioned medicinal species in *the Encyclopedia*, seven were introduced (*Dysphania ambrosioides*, *Taraxacum campylodes*, *Plantago major*, *Ruta graveolens*, *Borago officinalis*, *Zingiber officinale*, and *Sonchus oleraceus*) [33]. Introduced species were most represented in Asteraceae, Fabaceae, Poaceae, Lamiaceae and Rosaceae.

**Table 1:** Contingency table for test of non-independence between plant origin and use as a medicinal.

	Non-medicinal	Medicinal
**Native**	13174	1984
**Introduced**	394	201

Medicinal status was not independent of plant origin (χ^2^ = 203.48 df = 1, p < 0.0001).

### Availability hypothesis: Accessibility of plants for use

The best model for medicinal plant use included all predictors (origin, cultivation and number of provinces of occurrence), and also the interaction between origin and number of provinces of occurrence, providing support for the availability hypothesis. A plant was more likely to be medicinal if it was introduced, if it was cultivated and if it occurred across a larger number of provinces. Occurrence over a larger number of provinces had a stronger influence on the chances of an introduced plant’s being medicinal compared to a native plant (β = 0.25 ± 0.11, z = 2.19, p = 0.028). Cultivation was the strongest predictor of medicinal use of a plant (β = 1.11 ± 0.14 [SE], z = 7.8, p < 0.0001; odds ratio 2.95 [2.24; 3.88]), followed by the number of provinces of occurrence (β = 0.80 ± 0.02, z = 37.2, p < 0.0001; odds ratio 2.19 [1.55; 2.64]), and plant origin (β = 0.68 ± 0.14, z = 4.9, p < 0.0001; odds ratio 2.02 [0.10; 0.11]).

When cultivated plants were removed from this model, introduced status (β = 0.92 ± 0.17, z = 5.5, p < 0.0001) and number of provinces of occurrence (β = 0.80± 0.02, z = 36.9, p < 0.0001) were still significant predictors for a plant’s medicinal status. The interaction of plant origin and number of provinces of occurrence was not significant (β = 0.07 ± 14.9, z = 0.49, p = 0.63), meaning there was no detected difference in how availability by number of provinces influenced the probability of medicinal use for wild or naturalized introduced and native plants.

**Fig 1:**
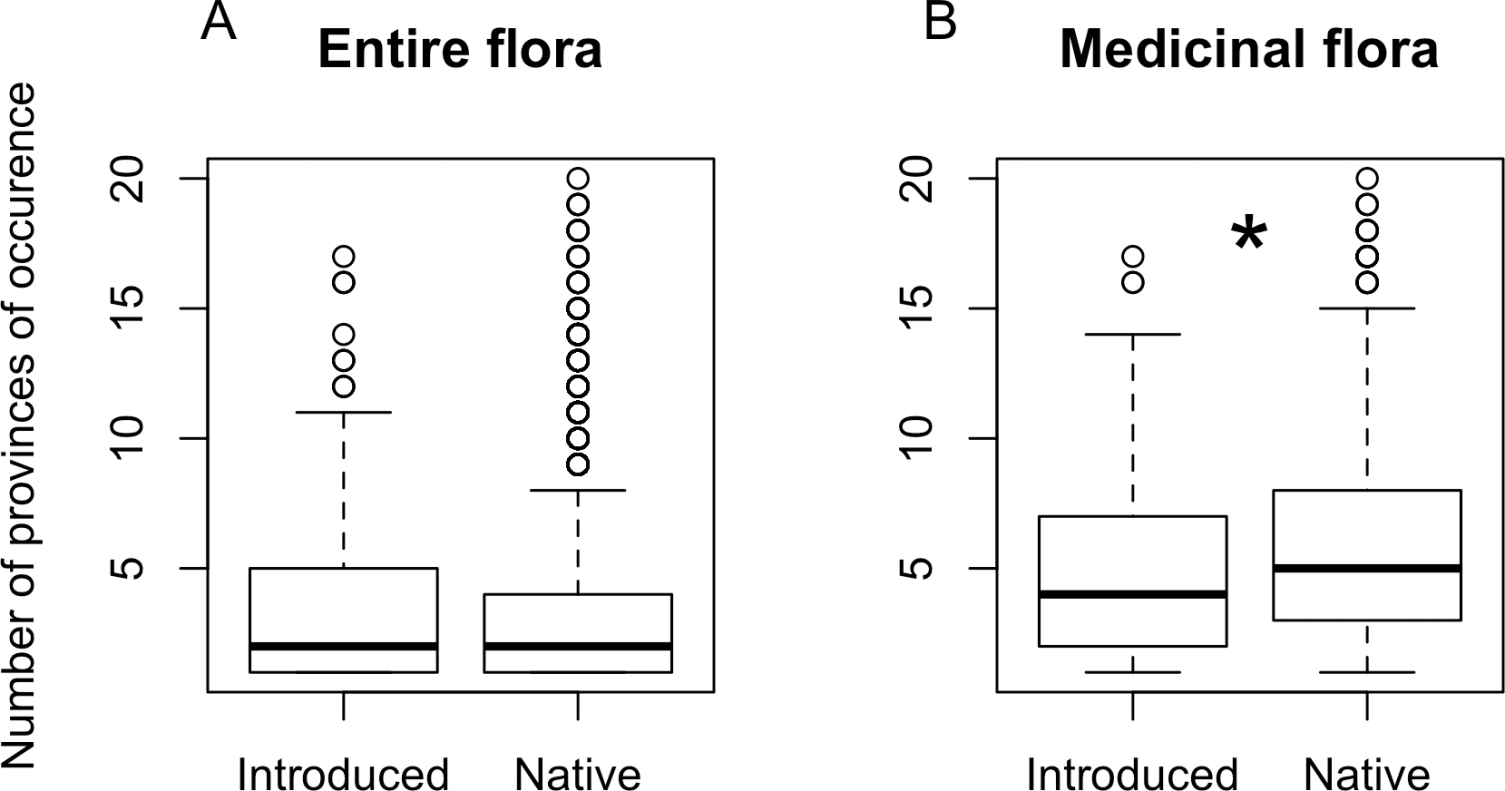
Number of provinces of occurrence for native and introduced plants. (A) Among the overall Ecuadorian flora (n = 15161 and 595) (χ^2^ = 0.0076, df = 1, p = 0.93), and (B) among medicinal plants (n = 1843 and 189)(χ^2^ = 8.29, df = 1, p = 0.004).

Most introduced species (58%) and just less than 1% of native plants in Ecuador are cultivated (χ^2^ = 6212, df = 1, p < 0.001). Introduced plants in the overall flora did not differ from native plants in the number of provinces of occurrence (Kruskal-Wallis χ^2^ = 0.0076, df = 1, p = 0.93, Fig 1A). For the subset of this overall flora that is used medicinally, introduced species were twenty-times more likely to be cultivated as compared to native medicinals (66% introduced medicinal species are cultivated compared to 3.3% of native medicinals, χ^2^ = 815.12, df = 1, p < 0.001). Introduced medicinal plants were less widespread than native medicinals in the number of provinces of occurrence (Kruskal-Wallis χ^2^ = 8.29, df = 1, p = 0.004, Fig 1B).

### Diversification hypothesis: Therapeutic applications

Plant origin makes a significant contribution to explaining the number of species that treat a specific body system (40 units reduction in AIC compared to model with interaction term, see Methods) or a specific medical condition (50 units reduction in AIC with removal of interaction term). Introduced plants tend to be most represented in the treatment of circulatory, digestive, respiratory, blood, endocrine and metabolic systems disorders (Table 2). Native plants tend to be most represented as anesthetics, antidotes, and for treatment of cuts and wounds (Table 3).

**Table 2:** Number of introduced and native plants that are employed to treat each body system.

Body System	Introduced	Native
Integumentary system disorders	42	275
Digestive system disorders	108	262
Endocrine system disorders	34	89
Skeleto-muscular system disorders	44	157
Immune system disorders	1	22
Metabolic system disorders	13	24
Respiratory system disorders	74	218
Urogenital system disorders	78	166
Circulatory system disorders	53	100
Nervous system disorders	53	172

**Table 3:** Number of introduced and native plants that are employed for each treatment category.

Treatment Category	Introduced	Native
Anesthetic	0	14
Antidote	16	324
Mental disorders	12	26
Non-specified disorders	110	723
Nutritional disorders	17	42
Cuts and wounds	54	366
Infections or infestations	107	553
Inflammation	61	275
Cancer and tumors	17	85

**Fig 2:**
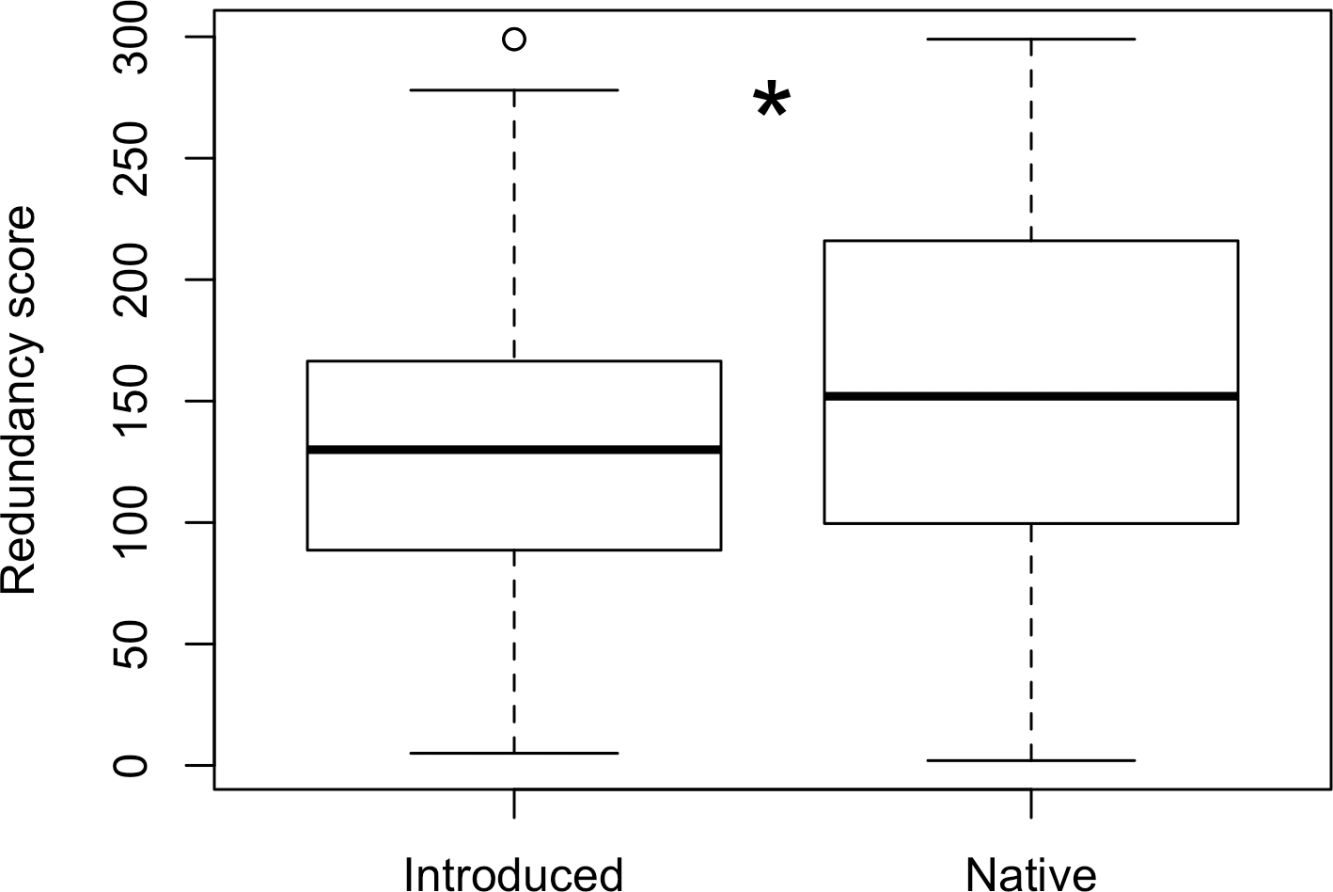
Redundancy score for introduced and medicinal native species. Wilcoxon rank sum test (W = 243420, p < 0.0001).

Fewer introduced plant species treated Old World diseases compared to diseases present prior to the Spanish Conquest (Tables 4 and 5, χ^2^ = 7.38, df = 1, p = 0.007). Introduced medicinal species made up 28% of species used to treat pre-Contact diseases and 16% of the species used to treat post-Contact diseases, or “Old World” diseases. Introduced species were less medically redundant than native plants (W = 243420 p < 0.0001, Fig 2) suggesting that introduced plants are utilized for diseases with fewer native plant treatment options.

**Table 4:** Number of native and introduced plants that treat post-Contact (Old World) and pre-Contact diseases.

Disease or Syndrome	Arrival of disease	Native	Introduced
Smallpox	post-Contact	10	1
Measles	post-Contact	19	7
Malaria	post-Contact	40	0
Chickenpox	post-Contact	1	0
Dysentery	post-Contact	24	7
Influenza	post-Contact	6	4
Cholera	post-Contact	1	0
Hepatitis	post-Contact	8	2
Mumps	post-Contact	6	1
Cancer	pre-Contact	75	19
Pneumonia	pre-Contact	23	9
Bronchitis	pre-Contact	20	14
Common cold	pre-Contact	92	44
Arthritis	pre-Contact	62	42
Leishmaniasis	pre-Contact	2	0
Dementia	pre-Contact	2	2
Asthma	pre-Contact	28	17
Stomach flu	pre-Contact	101	25
Scurvy	pre-Contact	10	3
Herpes	pre-Contact	8	3
Gangrene	pre-Contact	16	4
Scabies	pre-Contact	49	11
Colerín	pre-Contact	15	9
Conjunctivitis	pre-Contact	4	3
Holanda	pre-Contact	21	2
Allergies	pre-Contact	13	0
Tabardillo	pre-Contact	5	1
Erysipelas	pre-Contact	8	3
Neurasthenia	pre-Contact	2	0

**Table 5:** Contingency table to test if introduced plants are more likely to treat introduced (post Spanish contact) diseases.

	Native plants	Introduced plants
**Pre-Contact**	556	211
**Post-Contact**	115	22

Introduced plants were less likely to treat post-Contact diseases (χ^2^ = 7.38, df = 1, p = 0.007).

### Versatility hypothesis: Number of plant uses

**Fig 3:**
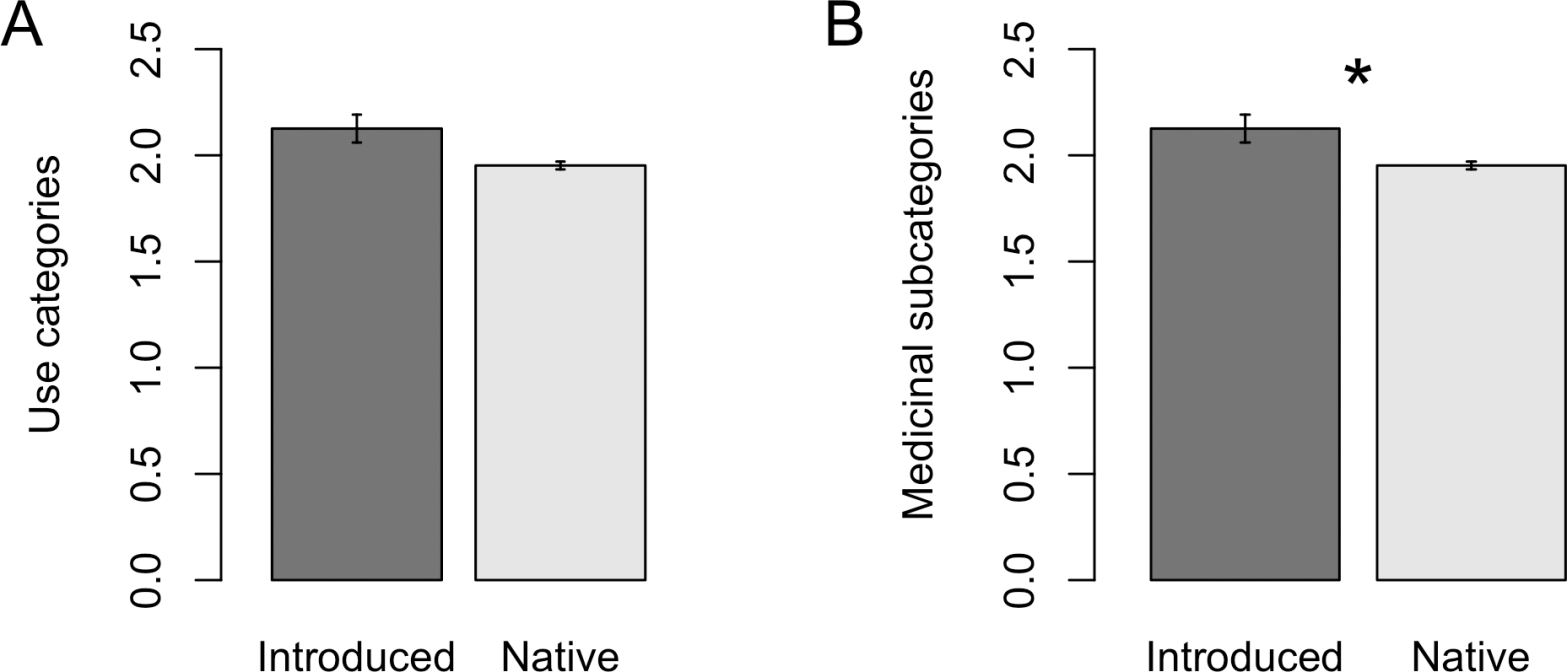
Average number of use categories and medicinal treatment subcategories per plant. (A) Use categories and (B) medicinal treatment subcategories, for introduced (n = 532 and 312) native (n = 4502 and 2230) plants. Error bars ± 2SE. Asterisk indicates significance (p < 0.05).

The number of use categories per plant did not differ between native and introduced species (Kruskal-Wallis χ^2^ = 5.68, df = 3, p = 0.13, Fig 3A), suggesting that introduced species were not significantly more versatile than native species across broad use categories. However, introduced species were more likely to be used for a greater number of medicinal treatment categories than were native species (Kruskal-Wallis χ^2^ = 48.8, df = 3, p < 0.001, Fig 3B).

## Discussion

The databases we used have certain biases that need to be considered in interpreting these results (see below), but they are unlikely to seriously affect the main results. Introduced plants were more likely to be used for medicine than would be predicted by chance. The majority of tested factors jointly help explain the over-representation of introduced plants in the pharmacopoeia, including greater availability at a province scale, stronger association with cultivation and greater usefulness based on diversification and versatility. For diversification, we find support based on use categories and redundancy, but not for treatment of introduced diseases. For versatility, we find support for therapeutic versatility, but not versatility of use categories.

### Availability through cultivation associated with over-representation of introduced medicinal plants

The over-representation of introduced plants as medicine in our study could also result, in part, from a tendency of studies in *the Encyclopedia* to emphasize collection in markets and homegardens. This may have increased the chance of identifying medicinal uses for introduced compared to native plants, as many cultivars and commercialized plants are introduced.

We found consistent support for the availability hypothesis when considering the Ecuadorian pharmacopoeia as a whole. When analyzing patterns for introduced compared to native plants, we found that availability based on the number of provinces of occurrence was a stronger predictor of medicinal use for introduced plants. The stronger importance of geographic distribution for introduced plants was observed despite the fact that distribution could have been under-estimated for introduced plants in our data, as introduced plants are likely under-collected in studies used in the compilation of *the Catalogue*. However, when cultivated species were removed from the analysis, there was no longer a significant difference between introduced and native plants with regard to the impact of number of provinces of occurrence on medicinal use. This suggests that the spread of introduced and cultivated species across provinces explains the above finding that the number of provinces of occurrence was more important in explaining medicinal use of introduced compared to native plants. However, given that many introduced weedy medicinal species are also cultivated, the disproportionate association of introduced species with widespread anthropogenic habitats could also explain their disproportionate use in medicine compared to native plants.

Of the three predictors of medicinal plant use tested in our model, cultivation was the strongest: a cultivated plant was nearly three-times as likely as a non-cultivated plant to be used medicinally. The strong association of medicinal use and cultivation is expected given that people cultivate useful plants to make them accessible, and newly accepted medicinal plants are often those that are already under cultivation in other regions. The latter may be the strongest factor influencing our data, given the discussion above, and given the association of introduced plants with cultural diffusion [42] and the large Mestizo population in Ecuador that utilizes plant medicine. It is unclear how many of the cultivated medicinal plants in Ecuador were cultivated because they are medicinal, and how many had their medicinal use discovered after they were already cultivated for other purposes, such as for food [43]. We cannot, and do not aim to, identify cultivation as a factor *causing* medicinal use. However, the strong association of cultivation with medicinal use highlights the importance of further investigation into the types of cultivation and the relationship of cultivation to availability for future theoretical development of the availability hypothesis. Questions associated with the development of the availability hypothesis are, for example: when are wild plants preferred despite availability of the same cultivated species?, how has global change influenced decisions to cultivate medicinal plants?, and when does cultivation increase or not the access to plants, and for whom?

The over-representation of introduced plant species as medicine in this study is consistent with results from [24] on weedy species used by the Highland Maya of Chiapas, Mexico, and in Native North America, as well as with results for introduced species by other studies in the region [44]. Stepp and Moerman [24] explained the disproportionately high use of weeds as medicine based on their availability and chemistry. These authors suggest that people prefer to use medicines that are relatively easy to acquire. Weeds tend to grow in anthropogenic habitats closer to people’s homes, and weeds tend to be fast-growing herbs that have low-weight qualitative defense compounds effective as medicines [45]. While these factors may apply to Ecuador—a large proportion of the medicinal plants reported in Ecuador are herbaceous [33] and grow successfully in disturbed areas [46]—cultivation also needs to be carefully considered as a factor impacting and potentially impacted by availability.

In addition to geographic distance, availability in terms of local land tenure rights [47], selling price [30] and seasonality [29] could play important roles in use patterns. Perennial plants would be given preference according to the seasonality hypothesis, as they are more likely to be available, providing greater medicinal security [29,48], though this is unlikely to be an important factor in Ecuador where seasonality is less pronounced. Prestige, palatability and efficacy are additional factors that have been considered in community acceptance of new plants for medicine [4,30]. The scale and type of availability are also important. A meta-analysis of medicinal plant use in NE Brazil revealed that more native plants are utilized at the cumulative regional level, but not at the local level [49].

Finally, the northern Andes are a biodiversity hotspot considered a priority for biodiversity conservation [50]. Regional loss of biodiversity resulting from climate change and other social and economic factors could be driving some of the adoption of introduced species as medicine, as natives become less available [51].

### Introduced plants as a diversification strategy for local pharmacopoeia

Introduced plants made different treatment contributions than native plants by body system and treatment category (Tables 2 and 3), lending support to the possibility that these species were added to broaden the spectrum of treatment options. This is consistent with the diversification hypothesis [29,31]. For example, in the Caatinga region of Brazil, exotic species were the only plants that treated digestive problems, headaches and fevers [29]. Soldati and Albuquerque [22], in the same region, found 14 local therapeutic categories were treated only with exotic species. A follow-up study demonstrated that the difference in application between native and exotic species could have a chemical basis: native and exotic plants had significantly different occurrences of chemical compounds [31]. While we do not have specific information on bioactive compounds of medicinal plants in Ecuador, some of this information could be acquired from literature, or could be evaluated through a proxy such as phylogenetic diversity [52]. Given that introduced plants evolved in a region outside of Ecuador it is possible they could have phytochemistry distinctive from native plants in Ecuador.

In our study we found that introduced plants were less likely to treat post-Contact diseases than pre-Contact diseases (Tables 4 and 5). Similar findings are also reported by Bussmann and Sharon [44] in northern Peru and southern Ecuador. This consistent trend may reflect the timing of the introduction of the plant species analyzed (perhaps some plants were introduced well after the Spanish Conquest and, therefore, could not have been employed for those diseases, at least initially). It is also possible that introduced plants provided less effective treatments, or that in the social disruption of Contact and its attendant disease epidemics, little time and energy were available for medicinal experimentation [44,53]. Another possibility is that disease severity plays a role in our findings. Perhaps for more severe or life-threatening conditions, which tend to be the post-Contact diseases (e.g. [38,54]), native plants are utilized because they are better known and trusted due to their longer history of use. Finally, it should be noted that many Old World diseases are transmissible, and this may also have influenced our findings. The relationship of disease origin to selection of medicinal plant by origin has been only minimally investigated, and would be worthy of further study.

In Ecuador, introduced medicinal plants had lower redundancy scores, indicating that they were more likely to treat conditions for which there were fewer treatment options (Fig 2). This supports the hypothesis that introduced plants fill therapeutic vacancies. Utilitarian redundancy, however, is not a perfect evaluation of redundancy—multiple plants are often used in the preparation of traditional medicines, and alternative plants may not all treat the same condition with the same efficacy or in the same cultural or geographic context. Redundancy scores do, however, provide a useful starting point for further study.

### The role of plant broad utilitarian versatility versus medicinal therapeutic versatility

Our results indicate that introduced medicinal plants do not vary from natives in the average number of use categories (food, medicine, ornamental, etc.), but that they do treat a larger number of medical conditions. The greater versatility of introduced plant medicines, in terms of conditions treated, may be the result of direct selection by healers or communities for more versatile plants [30]. It could also be a consequence of the cultivated and cosmopolitan status of many introduced plant medicines. Being more cosmopolitan or more widespread geographically could increase versatility of introduced plants by increasing the opportunities for experimentation and, therefore, the probability of discovery of additional medicinal applications. A study of medicinal use of *Aloe* spp. in Kenya, for example, determined that the most widespread species, *Aloe secundiflora*, was also the aloe species with the most recorded uses [55].

### Holistic investigation of medicinal plant selection

There are limitations to understanding plant use patterns by humans based on counting numbers of species, and using large, heterogeneous datasets. Even if fewer introduced species were utilized, the frequency with which each plant is utilized is important to consider, as is the efficacy of each plant [4,25,56]. The frequency of application could be determined through ethnographic study within particular communities. In addition, concepts of diseases and health vary across cultures and communities and those in traditional societies, including many in Ecuador, do not necessarily fit the categories of western medicine [57,58]. For example, spiritual or “magical” uses can be quite important: “magic” was the most common category of ailment recorded for the use of medicinal plants in northern Peru and southern Ecuador [44]. Further, we recommend similar analyses from other regions to provide primary data for future meta-analysis that will draw global conclusions about the importance of introduced species in local pharmacopoeia.

To fully understand use patterns of plants for medicine by local and Indigenous Peoples, findings must be considered in the historic and contemporary cultural, political, economic and spiritual context of particular communities. For example, taboos, local stories, values and beliefs, and personal, family or community preferences and experiences could inform plant selection in ways that may or may not correspond to the values of efficacy, efficiency, and maximizing treatment options that are assumed in the hypotheses developed and tested here. Unfortunately, with urbanization, improvements in transportation, and changes in transmission of traditional knowledge, opportunities to acquire such local background knowledge are becoming rarer.

## Conclusion

Analysis of the two databases suggests that introduced plants are over-represented in the Ecuadorian pharmacopoeia. The disproportionate use of introduced plants in medicine was partially explained by all three tested hypotheses. The *availability hypothesis* was supported in most cases. Plant medicinal use was positively associated with introduced status as well as availability in terms of cultivation and geographic distribution, with cultivation showing the strongest association with medicinal use. Geographic distribution increased the probability of medicinal use more quickly for introduced compared to native plants, suggesting accessibility is more important in determining medicinal use for introduced compared to native plants. In terms of the *versatility hypothesis*, introduced medicinal plants treated more diseases, but did not have more broad use categories than native medicinal plants. The potential for introduced plants to meet unmet medicinal needs (*diversification hypothesis*) was supported by the lower redundancy score for introduced compared to native medicinal plants, and by their different spectrum of body system and disease treatment, but not in the treatment of introduced diseases.

This study highlights the nuanced way in which multiple hypotheses contribute to explaining plant use patterns. This work also reinforces the importance of the maintenance of human-plant relationships across the spectrum of cultivation practices—in community gardens, homegardens, farms and agroforestry settings—as these are important anthropogenic habitats for the provisioning of cultural and medicinal services to local people, and their importance is likely to increase with global change. Further study could measure and directly compare the source and therapeutic contribution of particular introduced medicinal plants in local communities as well as the role of cultivation, commercialization and cultural beliefs in shaping use patterns.

## Supporting information

S1 Table Introduced medicinal plants with cultivation status. (PDF)

S2 Table Richness of native and introduced species by province. (PDF)

S3 Table Treatment target categories and search criteria. (PDF)

S1 Text Spanish abstract. (PDF)
