# Supplementary material for: Availability, diversification and versatility explain human selection of introduced plants in Ecuadorian traditional medicine
Source: PLoS One. 2017 Sep 8;12(9):e0184369. doi: 10.1371/journal.pone.0184369 (PMC5590918; doi:10.1371/journal.pone.0184369)
Supplement: S1 Table — (PDF) [file pone.0184369.s001.pdf]

S1 Table. **Introduced medicinal plants with cultivation status.**

Family	Species	Author	Cultivation status
Acanthaceae	*Acanthus mollis*	L.	cultivated
Adoxaceae	*Sambucus nigra*	L.	cultivated
Amaranthaceae	*Aerva sanguinolenta*	(L.) Blume	cultivated
Amaranthaceae	*Beta vulgaris*	L.	cultivated
Amaranthaceae	*Chenopodium album*	L.	-
Amaranthaceae	*Dysphania ambrosioides*	(L.) Mosyakin & Clemants	cultivated
Amaranthaceae	*Rumex acetosella*	L.	cultivated
Amaranthaceae	*Rumex crispus*	L.	cultivated
Amaranthaceae	*Rumex longifolius*	D.C.	-
Amaranthaceae	*Rumex obtusifolius*	L.	cultivated
Amaranthaceae	*Spinacia oleracea*	L.	cultivated
Amaryllidaceae	*Allium cepa*	L.	cultivated
Amaryllidaceae	*Allium sativum*	L.	cultivated
Amaryllidaceae	*Crinum × amabile*	Donn ex Ker Gawl.	cultivated
Anacardiaceae	*Anacardium occidentale*	L.	-
Anacardiaceae	*Mangifera indica*	L.	cultivated
Anacardiaceae	*Schinus molle*	L.	cultivated
Anacardiaceae	*Spondias purpurea*	L.	cultivated
Annonaceae	*Guatteria schunkevigoi*	D.R. Simpson	-
Apiaceae	*Anethum graveolens*	L.	cultivated
Apiaceae	*Apium graveolens*	L.	cultivated
Apiaceae	*Coriandrum sativum*	L.	cultivated
Apiaceae	*Cyclospermum leptophyllum*	(Pers.) Sprague	-
Apiaceae	*Daucus carota*	L.	cultivated
Apiaceae	*Foeniculum vulgare*	Mill.	cultivated
Apiaceae	*Petroselinum crispum*	(Mill.) Fuss	cultivated
Apiaceae	*Pimpinella anisum*	L.	-
Apocynaceae	*Cascabela thevetia*	(L.) Lippold	cultivated
Apocynaceae	*Catharanthus roseus*	(L.) G.Don	cultivated
Apocynaceae	*Nerium oleander*	L.	cultivated
Apocynaceae	*Plumeria rubra*	L.	cultivated
Apocynaceae	*Vinca major*	L.	cultivated
Aquifoliaceae	*Ilex paraguariensis*	A.St.-Hil.	cultivated
Araceae	*Zantedeschia aethiopica*	(L.) Spreng.	cultivated
Arecaceae	*Bactris gasipaes*	Kunth	cultivated
Arecaceae	*Cocos nucifera*	L.	cultivated
Asparagaceae	*Agave americana*	L.	cultivated
Asparagaceae	*Asparagus officinalis*	L.	cultivated
Asparagaceae	*Cordyline fruticosa*	(L.) A.Chev.	cultivated
Asparagaceae	*Sansevieria trifasciata*	Prain.	cultivated
Asparagaceae	*Yucca aloifolia*	L.	cultivated
Asteraceae	*Achillea millefolium*	L.	-
Asteraceae	*Acmella oleracea*	(L.) R.K. Jansen	cultivated
Asteraceae	*Ageratum conyzoides*	(L.) L.	-
Asteraceae	*Artemisia absinthium*	L.	cultivated
Asteraceae	*Blainvillea acmella*	(L.) Philipson	-
Asteraceae	*Calendula officinalis*	L.	cultivated

Asteraceae	*Cotula australis*	(Sieber ex Spreng.) Hook.f.	-
Asteraceae	*Cynara cardunculus*	L.	cultivated
Asteraceae	*Emilia fosbergii*	Nicolson	-
Asteraceae	*Erechtites hieraciifolia*	(L.) Raf. ex DC.	cultivated
Asteraceae	*Erigeron bonariensis*	L.	-
Asteraceae	*Gnaphalium coarctatum*	Willd.	-
Asteraceae	*Hypochaeris radicata**	L.	-
Asteraceae	*Lactuca sativa*	L.	cultivated
Asteraceae	*Matricaria chamomilla*	L.	cultivated
Asteraceae	*Picrosia longifolia*	D.Don	-
Asteraceae	*Sigesbeckia serrata*	DC.	-
Asteraceae	*Silybum marianum*	(L.) Gaertn.	-
Asteraceae	*Soliva anthemifolia*	(Juss.) Sweet	-
Asteraceae	*Sonchus oleraceus*	(L.) L.	cultivated
Asteraceae	*Tagetes erecta*	L.	cultivated
Asteraceae	*Tanacetum parthenium*	(L.) Sch.Bip.	-
Asteraceae	*Taraxacum argutifrons*	A.J.Richards	-
Asteraceae	*Taraxacum campylodes*	G.E.Haglund	cultivated
Balsaminaceae	*Impatiens balsamina*	L.	cultivated
Balsaminaceae	*Impatiens noli-tangere*	L.	cultivated
Balsaminaceae	*Impatiens walleriana*	Hook.f.	cultivated
Basellaceae	*Ullucus tuberosus*	Caldas	cultivated
Bignoniaceae	*Crescentia cujete*	L.	cultivated
Bignoniaceae	*Jacaranda mimosifolia*	D.Don	cultivated
Boraginaceae	*Borago officinalis*	L.	cultivated
Boraginaceae	*Cynoglossum amabile*	Stapf & J.R.Drumm.	-
Boraginaceae	*Heliotropium indicum*	L.	-
Brassicaceae	*Brassica napus*	L.	cultivated
Brassicaceae	*Brassica nigra*	(L.) K.Koch	cultivated
Brassicaceae	*Brassica oleracea*	L.	cultivated
Brassicaceae	*Brassica rapa*	L.	cultivated
Brassicaceae	*Capsella bursa-pastoris*	(L.) Medik.	cultivated
Brassicaceae	*Erysimum x cheiri*	(L.) Crantz	cultivated
Brassicaceae	*Lobularia maritime*	(L.) Desv.	cultivated
Brassicaceae	*Matthiola incana*	(L.) R.Br.	cultivated
Brassicaceae	*Nasturtium officinale*	R.Br.	cultivated
Brassicaceae	*Raphanus raphanistrum*	L.	cultivated
Brassicaceae	*Sinapis arvensis*	L.	-
Brassicaceae	*Sisymbrium officinale*	(L.) Scop.	cultivated
Bromeliaceae	*Ananas comosus*	(L.) Merr.	cultivated
Cactaceae	*Cereus repandus*	(L.) Mill.	-
Calophyllaceae	*Mammea americana*	L.	cultivated
Campanulaceae	*Hippobroma longiflora*	(L.) G.Don	-
Cannabaceae	*Cannabis sativa*	L.	cultivated
Caprifoliaceae	*Dipsacus fullonum*	L.	cultivated
Caprifoliaceae	*Scabiosa atropurpurea*	L.	-
Caprifoliaceae	*Valeriana officinalis*	L.	-
Caryophyllaceae	*Dianthus caryophyllus*	L.	cultivated
Caryophyllaceae	*Silene gallica*	L.	-
Combretaceae	*Combretum indicum*	(L.) DeFilipps	cultivated
Combretaceae	*Terminalia catappa*	L.	cultivated

Commelinaceae	*Tradescantia spathacea*	Sw.	cultivated
Convolvulaceae	*Convolvulus arvensis*	L.	-
Convolvulaceae	*Ipomoea triloba*	L.	cultivated
Crassulaceae	*Bryophyllum pinnatum*	(Lam.) Oken	cultivated
Cucurbitaceae	*Citrullus lanatus*	(Thunb.) Matsum. & Nakai	cultivated
Cucurbitaceae	*Cucumis melo*	L.	cultivated
Cucurbitaceae	*Cucurbita ficifolia*	Bouché	cultivated
Cucurbitaceae	*Cucurbita maxima*	Duchesne	cultivated
Cucurbitaceae	*Cucurbita moschata*	Duchesne	cultivated
Cucurbitaceae	*Luffa cylindrical*	(L.) M.Roem.	cultivated
Cucurbitaceae	*Momordica charantia*	L.	cultivated
Cupressaceae	*Cupressus lusitanica*	Mill.	cultivated
Cupressaceae	*Cupressus macrocarpa*	Hartw.	cultivated
Dioscoreaceae	*Dioscorea bulbifera*	L.	cultivated
Equisetaceae	*Equisetum arvense*	L.	-
Euphorbiaceae	*Acalypha hispida*	Burm.f.	cultivated
Euphorbiaceae	*Croton leptostachyus*	Kunth	-
Euphorbiaceae	*Croton malambo*	H.Karst.	-
Euphorbiaceae	*Croton niveus*	Jacq.	-
Euphorbiaceae	*Croton tiglium*	L.	-
Euphorbiaceae	*Euphorbia cotinifolia*	L.	cultivated
Euphorbiaceae	*Euphorbia lathyris*	L.	-
Euphorbiaceae	*Euphorbia milii*	Des Moul.	cultivated
Euphorbiaceae	*Euphorbia peplus*	L.	cultivated
Euphorbiaceae	*Euphorbia tirucalli*	L.	cultivated
Euphorbiaceae	*Hura crepitans*	L.	cultivated
Euphorbiaceae	*Jatropha curcas*	L.	cultivated
Euphorbiaceae	*Manihot esculenta*	Crantz	cultivated
Euphorbiaceae	*Ricinus communis*	L.	cultivated
Fabaceae	*Arachis hypogaea*	L.	cultivated
Fabaceae	*Caesalpinia pulcherrima*	(L.) Sw.	cultivated
Fabaceae	*Cajanus cajan*	(L.) Millsp.	cultivated
Fabaceae	*Cassia fistula*	L.	cultivated
Fabaceae	*Cicer arietinum*	L.	cultivated
Fabaceae	*Clitoria ternatea*	L.	cultivated
Fabaceae	*Erythrina mitis*	Jacq.	-
Fabaceae	*Gliricidia sepium*	(Jacq.) Walp.	cultivated
Fabaceae	*Medicago polymorpha*	L.	-
Fabaceae	*Medicago sativa*	L.	cultivated
Fabaceae	*Melilotus indicus*	(L.) All.	-
Fabaceae	*Pisum sativum*	L.	cultivated
Fabaceae	*Senna alata*	(L.) Roxb	cultivated
Fabaceae	*Senna cernua*	(Balb.) H.S.Irwin & Barneby	-
Fabaceae	*Senna holosericea*	(Fresen.) Greuter	-
Fabaceae	*Spartium junceum*	L.	cultivated
Fabaceae	*Tamarindus indica*	L.	cultivated
Fabaceae	*Trifolium pratense*	L.	cultivated
Fabaceae	*Trifolium repens*	L.	cultivated
Fabaceae	*Vicia faba*	L.	cultivated
Gentianaceae	*Centaurium erythraea*	Rafn.	-

Geraniaceae	*Erodium cicutarium*	(L.) L'Hér.	-
Geraniaceae	*Erodium moschatum*	(L.) L'Hér.	-
Geraniaceae	*Pelargonium condensatum**	(Pers.) Poir.	-
Geraniaceae	*Pelargonium graveolens*	L'Hér.	cultivated
Geraniaceae	*Pelargonium odoratissimum*	(L.) L'Hér.	cultivated
Geraniaceae	*Pelargonium peltatum*	(L.) L'Hér.	cultivated
Geraniaceae	*Pelargonium vitifolium*	(L.) L'Hér.	cultivated
Hydrangeaceae	*Hydrangea macrophylla*	(Thunb.) Ser.	cultivated
Hyperiaceae	*Hypericum montanum*	L.	-
Hypericaceae	*Hypericum canadense*	L.	-
Icacinaceae	*Pleurisanthes artocarpi*	Baill.	-
Iridaceae	*Iris x germanica*	L.	-
Iridaceae	*Trimezia martinicensis*	(Jacq.) Herb.	-
Juglandaceae	*Juglans regia*	L.	cultivated
Lamiaceae	*Lavandula angustifolia*	Mill.	cultivated
Lamiaceae	*Marrubium vulgare*	L.	-
Lamiaceae	*Melissa officinalis*	L.	cultivated
Lamiaceae	*Mentha pulegium*	L.	cultivated
Lamiaceae	*Mentha spicata*	L.	cultivated
Lamiaceae	*Mentha suaveolens*	Ehrh.	cultivated
Lamiaceae	*Mentha x piperita*	L.	cultivated
Lamiaceae	*Ocimum basilicum*	L.	cultivated
Lamiaceae	*Origanum majorana*	L.	cultivated
Lamiaceae	*Origanum vulgare*	L.	cultivated
Lamiaceae	*Origanum x majoricum*	Cambess.	cultivated
Lamiaceae	*Rosmarinus officinalis*	L.	cultivated
Lamiaceae	*Salvia coccinea*	Buc'hoz ex Etl.	-
Lamiaceae	*Salvia hispanica*	L.	-
Lamiaceae	*Salvia officinalis*	L.	cultivated
Lamiaceae	*Stachys byzantina*	K.Koch	cultivated
Lamiaceae	*Thymus vulgaris*	L.	cultivated
Lauraceae	*Cinnamomum camphora*	(L.) J.Presl	-
Liliaceae	*Lilium longiflorum*	Thunb.	cultivated
Linaceae	*Linum usitatissimum*	L.	cultivated
Linderniaceae	*Lindernia diffusa*	(L.) Wettst.	-
Lythraceae	*Lagerstroemia indica*	L.	cultivated
Lythraceae	*Lawsonia inermis*	L.	cultivated
Lythraceae	*Punica granatum*	L.	-
Malvaceae	*Abelmoschus moschatus*	Medik.	cultivated
Malvaceae	*Alcea rosea*	L.	cultivated
Malvaceae	*Althaea officinalis*	L.	cultivated
Malvaceae	*Hibiscus acetosella*	Welw. ex Hiern	cultivated
Malvaceae	*Hibiscus radiatus*	Cav.	cultivated
Malvaceae	*Hibiscus rosa-sinensis*	L.	cultivated
Malvaceae	*Malva arborea*	(L.) Webb & Berthel.	cultivated
Malvaceae	*Malva multiflora*	(Cav.) Soldano, Banfi & Galasso	cultivated
Malvaceae	*Malva parviflora*	L.	cultivated
Malvaceae	*Malva pusilla*	Sm.	cultivated

Malvaceae	*Malva sylvestris*	L.	cultivated
Malvaceae	*Theobroma bicolor*	Humb. & Bonpl.	cultivated
Meliaceae	*Azadirachta indica*	A.Juss.	cultivated
Meliaceae	*Melia azedarach*	L.	cultivated
Moraceae	*Artocarpus altilis*	(Parkinson ex F.A.Zorn) Fosberg	cultivated
Moraceae	*Ficus carica*	L.	cultivated
Musaceae	*Musa acuminate*	Colla	cultivated
Musaceae	*Musa x paradisiaca*	L.	cultivated
Myrtaceae	*Corymbia citriodora*	(Hook.) K.D.Hill & L.A.S.Johnson	cultivated
Myrtaceae	*Eucalyptus globulus*	Labill.	cultivated
Myrtaceae	*Myrtus communis*	L.	-
Myrtaceae	*Syzygium jambos*	(L.) Alston	cultivated
Nyctaginaceae	*Bougainvillea spectabilis*	Willd.	cultivated
Nyctaginaceae	*Mirabilis jalapa*	L.	cultivated
Oleaceae	*Jasminum grandiflorum*	L.	cultivated
Oleaceae	*Jasminum sambac*	(L.) Aiton	cultivated
Oleaceae	*Olea europaea*	L.	-
Onagraceae	*Fuchsia hybrida*	hort. ex Siebert & Voss	cultivated
Onagraceae	*Fuchsia magellanica*	Lam.	cultivated
Papaveraceae	*Argemone mexicana*	L.	cultivated
Papaveraceae	*Chelidonium majus*	L.	cultivated
Papaveraceae	*Fumaria parviflora*	Lam.	cultivated
Papaveraceae	*Papaver rhoeas*	L.	cultivated
Papaveraceae	*Papaver somniferum*	L.	cultivated
Passifloraceae	*Passiflora edulis*	Sims	cultivated
Phyllanthaceae	*Phyllanthus urinaria*	L.	-
Pinaceae	*Pinus radiata*	D.Don	cultivated
Plantaginaceae	*Digitalis purpurea*	L.	cultivated
Plantaginaceae	*Plantago afra*	L.	-
Plantaginaceae	*Plantago lanceolata*	L.	-
Plantaginaceae	*Plantago major*	L.	cultivated
Poaceae	*Avena fatua*	L.	cultivated
Poaceae	*Briza minor*	L.	-
Poaceae	*Coix lacryma-jobi*	L.	cultivated
Poaceae	*Cymbopogon citratus*	(DC.) Stapf	cultivated
Poaceae	*Cymbopogon schoenanthus*	(L.) Spreng.	-
Poaceae	*Cynodon dactylon*	(L.) Pers.	-
Poaceae	*Digitaria setigera*	Roth	-
Poaceae	*Eleusine indica*	(L.) Gaertn.	-
Poaceae	*Holcus lanatus*	L.	-
Poaceae	*Hordeum vulgare*	L.	cultivated
Poaceae	*Lolium temulentum*	L.	-
Poaceae	*Melinis minutiflora*	P.Beauv.	-
Poaceae	*Oryza sativa*	L.	cultivated
Poaceae	*Panicum maximum*	Jacq.	cultivated
Poaceae	*Pennisetum clandestinum*	Hochst. ex Chiov.	cultivated
Poaceae	*Phalaris angusta*	Nees ex Trin.	-
Poaceae	*Poa annua*	L.	-

Poaceae	*Saccharum officinarum*	L.	cultivated
Poaceae	*Vulpia bromoides*	(L.) Gray	-
Poaceae	*Zea mays*	L.	cultivated
Polygonaceae	*Fagopyrum esculentum*	Moench	-
Polygonaceae	*Persicaria punctata*	(Elliott) Small	-
Polygonaceae	*Rumex acetosa*	L.	cultivated
Ranunculaceae	*Consolida ajacis*	(L.) Schur	-
Rosaceae	*Chaenomeles sinesise*	(Dum.Cours.) Koehn	cultivated
Rosaceae	*Cydonia oblonga*	Mill.	-
Rosaceae	*Eriobotrya japonica*	(Thunb.) Lindl.	cultivated
Rosaceae	*Fragaria chiloensis*	(L.) Mill.	cultivated
Rosaceae	*Fragaria vesca*	L.	cultivated
Rosaceae	*Mespilus germanica*	L.	cultivated
Rosaceae	*Prunus armeniaca*	L.	-
Rosaceae	*Prunus domestica*	L.	cultivated
Rosaceae	*Prunus persica*	(L.) Batsch	cultivated
Rosaceae	*Rosa alba*	L.	cultivated
Rosaceae	*Rosa centifolia*	L.	-
Rosaceae	*Rosa cymosa*	Tratt.	cultivated
Rosaceae	*Rubus niveus*	Thunb.	cultivated
Rosaceae	*Rubus vestitus*	Weihe	-
Rosaceae	*Sanguisorba minor*	Scop.	cultivated
Rubiaceae	*Coffea arabica*	L.	cultivated
Rubiaceae	*Coffea canephora*	Pierre ex A.Froehner	cultivated
Rutaceae	*Citrus maxima*	(Burm.) Merr.	cultivated
Rutaceae	*Citrus medica*	L.	cultivated
Rutaceae	*Citrus reticulata*	Blanco	cultivated
Rutaceae	*Murraya paniculata*	(L.) Jack	cultivated
Rutaceae	*Ruta graveolens*	L.	cultivated
Sapotaceae	*Pouteria sapota*	(Jacq.) H.E.Moore & Stearn	cultivated
Scrophulariaceae	*Verbascum phlomoides*	L.	-
Smilacaceae	*Smilax aspera*	L.	-
Smilacaceae	*Smilax glauca*	Walter	-
Smilacaceae	*Smilax laurifolia*	L.	-
Solanaceae	*Lycianthes stellata*	(Jacq.) Bitter	-
Solanaceae	*Nicandra physalodes*	(L.) Gaertn.	-
Solanaceae	*Nicotiana tabacum*	L.	cultivated
Thelypteridaceae	*Christella dentata*	(Forssk.) Brownsey & Jermy	cultivated
Tropaeolaceae	*Tropaeolum majus*	L.	cultivated
Urticaceae	*Urtica dioica*	L.	-
Urticaceae	*Urtica urens*	L.	-
Verbenaceae	*Lantana camara*	L.	cultivated
Violaceae	*Viola arvensis*	Murray	-
Violaceae	*Viola odorata*	L.	cultivated
Violaceae	*Viola tricolor*	L.	-
Vitaceae	*Vitis vinifera*	L.	cultivated
Xanthorrhoeaceae	*Aloe arborescens*	Mill.	-
Xanthorrhoeaceae	*Aloe buhrii*	Lavranos	-
Xanthorrhoeaceae	*Aloe dichotoma*	Masson	-
Xanthorrhoeaceae	*Aloe ferox*	Mill.	-

Xanthorrhoeaceae	*Aloe plicatilis*	(L.) Mill.	-
Xanthorrhoeaceae	*Aloe spectabilis*	Reynolds	-
Xanthorrhoeaceae	*Aloe vera*	(L.) Burm.f.	cultivated
Zingiberaceae	*Alpinia purpurata*	(Vieill.) K.Schum.	cultivated
Zingiberaceae	*Hedychium coronarium*	J.Koenig	cultivated
Zingiberaceae	*Zingiber officinale*	Roscoe	cultivated
Zygophyllaceae	*Tribulus terrestris*	L.	-

*unresolved
