# Supplementary material for: Availability, diversification and versatility explain human selection of introduced plants in Ecuadorian traditional medicine
Source: PLoS One. 2017 Sep 8;12(9):e0184369. doi: 10.1371/journal.pone.0184369 (PMC5590918; doi:10.1371/journal.pone.0184369)
Supplement: S2 Table — (PDF) [file pone.0184369.s002.pdf]

S2 Table. Richness of native and introduced species by province.

Province	Native species	Introduced species
Azuay	1939	121
Bolívar	1272	65
Cañar	1082	55
Carchi	2921	73
Chimborazo	2040	99
Cotopaxi	1855	86
El Oro	1293	39
Esmeraldas	2324	63
Galapagos	692	131
Guayas	1600	127
Imbabura	2228	133
Loja	3040	123
Los Ríos	1698	155
Manabí	993	58
Morona-Santiago	3356	38
Napo	5881	116
Pastaza	3150	36
Pichincha	4732	301
Sucumbíos	1883	19
Tungurahua	2045	151
Zamora-Chinchipe	2722	12

Source: http://www.tropicos.org/Project/CE, searched 8.20.17
