# Supplementary material for: Availability, diversification and versatility explain human selection of introduced plants in Ecuadorian traditional medicine
Source: PLoS One. 2017 Sep 8;12(9):e0184369. doi: 10.1371/journal.pone.0184369 (PMC5590918; doi:10.1371/journal.pone.0184369)
Supplement: S3 Table — (PDF) [file pone.0184369.s003.pdf]

S3 Table. Treatment target categories and search criteria.

Treatment target	Search terms
Abscess	abscesos
Acne	granos|acné
Allergies and allergic reactions	reacciones alérgicas|alergias
Anemia	anemia
Analgesic	analgésico|dolores del cuerpo|dolor de cuerpo|dolor corporal|alivia los dolores de boca, de garganta y de muelas|dolor de la garganta|dolor de huesos|dolores en general|los dolores en general|el dolor del cuerpo|para tratar dolores
Anesthetic	anestésic
Arthritis	artri|reum
Asthma	asma
Bad urine	mal de orina
Birthing pain	dolor después del parto|el parto para tratar el dolor|dolores de parto|dolores del parto|dar a luz|dar la luz
Bladder infection	afecciones de la vejiga
Boils	forúnculos
Breast milk production	la producción de leche materna|galactogénico
Bronchitis	bronq|bronco
Burns	quemadura
Cancer/Tumor	cancer|cáncer|tumor
Chickenpox	varicela
Cholera	cólera|colera
Colerín	colerín
Common cold	resfr|catarro
Conjunctivitis	conjuntivitis|afecciones de los ojos
Constipation	laxante|estreñimiento
Contusion	golpes
Coolness	enfriamiento|sacar el frío|calentar el cuerpo|cuerpo esta frío|cuerpo está frío
Cough	tos|expectorante
Dementia	demencia|memoria
Diabetes	diabetes
Diarrhea	diarrea|antidiarréico|antidiarreico
Alcohol intoxication	la borrachera
Dysentery	disent|antidisentérico
Earache	dolor de oído

Eczema	eczema
Erysipelas	erisipela
Exhaustion	agotamiento|debilidad|consunción|debilitación|colapso|cansancio|fatiga
Fever	la fiebre|fiebre|antifebril|bajar la temperatura|reducir la temperatura corporal|febrífugo|bajar el calor
Flatulence	carminativ|carminativas|flatulencia
Flu	gripe|antigripal
Foot fungus	los hongos de pie
Gangrene	gangrena
Headache	dolor de cabeza|dolores de cabeza|jaqueca|migraña
Hemorrhage	hemorragia|detener el sangrado|hemorrágia
Hepatitis	hepatitis
Herpes	herpes
High blood pressure	bajar la presión alta
Holanda	la holanda
Impotence	impotencia|esterilidad|infecundidad
Inflammation	inflamacion|inflamación|hinchazon|demulcente|desinflamatorio|hinchazón|desinflamante|inflamacíones
Influenza	influencia|influenza
Insect bites	picadura de insectos
Insomnia	insomnio|para el sueño
Kidney pain and kidney infections	dolor de riñones|tratar los riñon|afecciones renales|afecciones del riñón|infección de los riñon
Leishmaniasis	lesh
Lice	piojos
Liver infection	hígado|afecciones hepáticas
Mal de ojo	mal de ojo|mal ojo|Mal de ojo
Malaria	malaria|paludismo
Measles	sarampi
Menstrual cramps	los cólicos menstruales
Mouth sores	ampollas en la boca
Mumps	paperas
Muscle spasms	antiespasmódica
Nasal congestion	congestión nasal
Nausea and vomiting	vómito|nausea|vomito|náusea
Nerve disorders	afecciones nerviosas
Neurasthenia	neurastenia
Parasites	parásitos intestinales|los parásitos|lombrices|vermifugo|vermífugo|desparasitante

Pneumonia	neumo|pulmonía
Postpartum recovery	efectos del parto|afecciones postparto|después del parto|afecciones post-parto
Purgative	purgante
Regulation of menstruation	regula la menstruación|adelantar la menstruación|flujo menstrual|regular la menstruación|emenagoga
Scabies	sarna
Scurvy	escorbuto
Skin fungus	hongos de la piel|empeines
Skin rash	sarpullido|salpullido
Smallpox	viruela
Venomous animal bite	mordeduras de serpiente|mordedura de serpiente|ataques de la serpiente|mordedura de la serpiente|la moderdura de serpientes|mordedura de culebra|modeduras de culebra|picaduras de alacrán|hormiga conga|hormigas conga
Spleen pain	dolor del bazo
Stomach ache	dolor de estómago|dolores estomacal|dolor estomacal|dolores de estómago|cólicos intestinales|dolencias digestivas|empacho|los cólicos de gases|cólico
Styes	orzuelos
Sudden cold air exposure	golpe de aire fr|aire frío|golpe de aire|la parálisis de la cara cuando se sale al aire|aire del fr
Syphilis	antisifilítico|la sífilis
Tabardillo (fever with similarities to Typhus fever)	tabardillo
Throat discomfort	molestias de la garganta|carraspera de la garganta|la ronquera
Tooth cavities	caries
Toothache	dolor de muela|dolor de diente
Tuberculosis	tuberculosis
Ulcers	úlceras
Urinary retention	no se puede orinar|paso de la orina|diurético|diuréticamente|diurética|diuretic
Wounds or lesions	herida|lesiones|lesión|cortadura|cortes|cicatrizante
excluded	desinfectante|estomátic|usan medicinal|usa medical|usa medicinal|usan medical|es medicinal|Es medicinal|afecciones indeterminadas

Notes: Vertical lines separate search terms. Abbreviated search terms are word stems that capture any subsequent characters. Phrases need exact matches.
