# Supplementary material for: Availability, diversification and versatility explain human selection of introduced plants in Ecuadorian traditional medicine
Source: PLoS One. 2017 Sep 8;12(9):e0184369. doi: 10.1371/journal.pone.0184369 (PMC5590918; doi:10.1371/journal.pone.0184369)
Supplement: S1 Text — (PDF) [file pone.0184369.s004.pdf]

S1 Text. Spanish abstract.

Resumen

En todo el mundo, la mayoría de personas usan plantas como una fuente primaria de atención sanitaria y las plantas introducidas se discuten cada vez más como medicina. La protección de este recurso para la salud humana depende de la comprensión de qué plantas se utilizan y de cómo sus patrones de uso cambiarán con el tiempo. El aumento del uso de plantas introducidas en las farmacopeas locales se ha explicado por su mayor abundancia o accesibilidad (hipótesis de la disponibilidad), su capacidad de curar desórdenes de salud que no son tratados por plantas nativas (hipótesis de la diversificación), o como un resultado de que las plantas introducidas tienen varios roles simultáneos distintos (hipótesis de la versatilidad). Con el fin de describir el rol de las plantas introducidas en Ecuador y de probar estas tres hipótesis, investigamos si las plantas introducidas están sobre-representadas en la farmacopea ecuatoriana y si su uso como plantas medicinales se explica mejor por la mayor disponibilidad de las plantas introducidas, distintas aplicaciones terapéuticas o un mayor número de categorías de uso.   A partir de 44 585 registros de uso y una lista de >17 000 especies de plantas del Ecuador, usamos inferencia de multimodelos para evaluar si más plantas introducidas se usan como medicina en Ecuador de las que se esperaría por azar y para examinar el sustento para cada una de las tres hipótesis mencionadas. Encontramos sustento para todas las hipótesis, aunque con matices. Se utilizan más plantas introducidas de las que se esperaría por azar, lo que puede explicarse por su área de distribución natural, por su fuerte relación con el cultivo, por su diversificación (excepto en el caso de enfermedades introducidas) y por su versatilidad, en términos del número de tratamientos medicinales que se le dan, pero no por el número de categorías de uso. Las plantas introducidas contribuyen de modo desproporcionadamente alto a la medicina tradicional basada en plantas en Ecuador. La fuerte relación entre el cultivo y el uso medicinal de las plantas introducidas destaca la importancia de mantener ambientes creados por el ser humano, como huertos caseros y sistemas agroforestales, para el aprovisionamiento de servicios de salud.
